# LMS-supported HyFlex clinical supervision model: Illuminating perspectives from teacher candidates in the department of English language teaching

**DOI:** 10.1016/j.heliyon.2024.e29503

**Published:** 2024-04-10

**Authors:** Ebru Melek Koc

**Affiliations:** Inonu University, Faculty of Education, Malatya, TR, Turkiye

**Keywords:** HyFlex, Clinical supervision, Mentoring, Teacher candidates, LMS, ELT

## Abstract

This study presents a HyFlex Clinical Supervision Model supported by a Learning Management System (LMS). The model is designed to facilitate a seamless transition from face-to-face to remote education, particularly in unforeseen circumstances such as the COVID-19 pandemic. The primary aim is to understand the mode preferences of teacher candidates for various aspects of the teaching process, including receiving feedback on lesson plans, structuring teaching sessions, observation, being observed, and receiving feedback after teaching sessions during mentoring activities. The study included 58 fourth-year teacher candidates who were enrolled in English Language Teaching departments across six state universities in five regions in Turkey. Data was collected through an online questionnaire using Google Forms and interviews conducted via Zoom. The study uncovered diverse preferences among teacher candidates with regards to teaching sessions, observation methods, and feedback. The HyFlex clinical supervision model proposed is adaptable to both traditional and remote practicum settings.

## 1 Introduction

Establishing a successful education system requires highly qualified and well-trained teachers. Effective pre-service education, aligned with teachers' specific branches, plays a crucial role in enabling them to fulfill their roles competently. Preservice teachers' ability to confidently apply the knowledge gained in their academic studies to professional settings relies on ample opportunities for practical experience before entering the workforce. The practicum is a crucial component of most teacher training programmes. It allows future teachers to apply theoretical knowledge and skills under the guidance of both school-based cooperating teachers and university supervisors [1,2].

The practicum aims to assess and guide preservice teachers in developing their competences [3]. Although practicum courses may vary in content among universities, their fundamental goal remains consistent: to evaluate the competences of teacher candidates and support their development. The triad members involved in the practicum process, namely the university supervisor, cooperating teacher, and teacher candidates, collaborate to monitor activities, provide feedback on lesson plans and delivery, and conduct assessments. In this mentoring process, experienced practitioners transfer their expertise to less experienced ones, with the cooperating teacher and university supervisor acting as mentors [4].

• Mentoring

Mentoring is a multifaceted process that involves shared learning and growth, fostering mutual benefit, interaction, and support for both parties involved [5]. In the specific context of teacher education [6], provide a more focused definition, describing mentoring as the one-to-one support of a teacher candidate by a more experienced teacher. This definition acknowledges the tailored guidance and support offered within the teacher education setting, emphasizing the importance of an experienced teacher guiding a novice through the complexities of the teaching profession.

[7] proposes a framework consisting of five factors for a mentoring model: personal attributes, system requirements, pedagogical knowledge, modelling, and feedback. Personal attributes refer to the essential personal and interpersonal skills that mentors possess to effectively engage with their mentees. These attributes underpin the ability to establish and maintain strong, trusting relationships, offering both professional guidance and emotional support to facilitate the mentee's development. System requirements pertain to the fundamental duties and obligations of mentors in offering extensive guidance and assistance to preservice teachers, particularly in navigating the complex educational landscape, comprehending school culture, adhering to curricular demands, and aligning teaching practices with state standards and student assessment protocols. Successful mentoring depends on the mentor demonstrating effective teaching practices and providing clear examples of both effective and ineffective strategies. This modelling, along with opportunities for mentees to actively observe and engage, is fundamental for their development in teaching, empowering them to implement these practices themselves. A mentor is responsible for exemplifying pedagogical approaches and addressing instructional challenges that mentees may not discern on their own. This significantly influences their journey towards professional competence. The mentor's role in providing feedback is pivotal and recognized as the most crucial aspect of their support for mentees. This entails providing constructive feedback that focuses on crucial aspects of teaching practice, including classroom management, assessment strategies, lesson planning, and resource preparation. Mentors deliver feedback both verbally and in writing, ensuring that it is specific, tactfully honest, and aimed at fostering the mentee's confidence, positive mindset, and instructional skills. Effective feedback necessitates that mentees are receptive to engaging in a reciprocal dialogue. Mentors observe and provide feedback to the mentee on their teaching, identifying strengths and areas for improvement. The feedback is tailored to encourage professional growth and improve educational outcomes [7].

Mentors are assigned various roles, and these roles may be perceived differently by teacher candidates and cooperating teachers. In her study [8], investigated the perceived roles of mentors for 1843 teacher candidates. The study revealed a range of responsibilities, including providing support, facilitating school orientation, offering moral support, providing feedback on lesson plans and teaching performance, guiding on teaching resources, participating in evaluation, self-preparing for the mentor role, giving feedback on observation forms, and offering written feedback. In a separate study carried out by [9], the views of 358 cooperating teachers on their roles as mentors were examined. The study found that seven out of ten roles were associated with ‘academic support,’ which was perceived as the predominant responsibility by cooperating teachers when compared to other roles. These roles included providing facilitative information to enhance classroom performance, giving constructive feedback on teaching performance, assisting teacher candidates in forming a professional identity and being aware of their professional development, supporting lesson planning, voluntarily offering facilitative information, and aiding in the use and understanding of observation forms.

The clinical supervision model typically follows a cyclical process comprising of five stages: pre-conference, observation, analysis, post-conference, and critique of the entire process [10]. During the pre-conference stage, the teacher candidate and the cooperating school mentor discuss the candidate's draft lesson plan, and the candidate revises the plan based on the feedback received. During the observation phase, the cooperating teacher and university mentor take notes on the teacher candidate's lesson. In the post-conference phase, the mentors identify the candidate's strengths and areas for improvement. Finally, the candidate, cooperating school teacher, and university mentor share general reflections, address any problems encountered during the lesson, and work together to develop solutions and strategies for improvement. Through this process, the mentor provides objective feedback, creates an action plan, and supports the teacher candidate in improving their teaching skills. This contributes to the ongoing development of teacher candidates' skills and knowledge [11].

• Traditional Clinical Supervision in the Study Context

In the context of the study, practical training in Turkey's fourth-year English as a Foreign Language (EFL) teacher education program consists of two applied courses. The School Experience course occurs in the fall semester, followed by the Teaching Practicum course in the spring semester, each led by a university instructor. Theoretical classes, led by university instructors, evaluate the activities of teacher candidates during practical sessions and discuss their experiences at cooperating schools. During the fall term, teacher candidates spend six hours per week for 14 weeks at a cooperating school under the supervision of an assigned English language teacher and a university supervisor, referred to as cooperating school mentor and university mentor, respectively, in this study. During this period, teacher candidates observe both the teaching sessions of the cooperating school mentor and their peers, and engage in micro-teaching sessions lasting 10–20 min. The cooperating school mentor provides feedback on the candidates' lesson plans before their teaching sessions, observes them, and guides post-lesson feedback sessions where the teacher candidates reflect on their experience. The university mentor visits the cooperating school, observes the teacher candidates in the classroom and participates in reflection meetings, conducting a total of two observations for each teacher candidate during the semester. During the practicum, the teacher candidates are evaluated collaboratively by the cooperating school mentor and university mentor. The same clinical supervisory process takes place in the spring term, but with the teacher candidates taking on more responsibility. Unlike the fall term, they teach for the entire class time every week. Additionally, the teacher candidate is assigned to another university mentor, cooperating school, and cooperating school mentor.

• Clinical Supervision Practices Worldwide During the Pandemic

Due to the Covid-19 pandemic, all educational institutions have suspended face-to-face education and have resorted to distance education as the only alternative. Higher education institutions have continued to provide instruction through various applications and tools. Universities have quickly adopted various distance education methods, including using their own distance education centres, learning management systems (LMS), and virtual classes integrated into these systems [12]. Similarly, primary, secondary, and high schools have also continued education during the pandemic using different modes, such as asynchronous, remote synchronous, or hybrid modes. Due to the remote implementation of the practicum process, preservice teachers have been unable to conduct school visits or engage in face-to-face lesson observation and teaching practice [13]. However, they have been able to carry out these activities on virtual classroom platforms.

The most effective method for supporting teacher education programs or field experiences, particularly practicum, does not involve remote learning and observations. Similarly, conducting practicum remotely has made it very difficult to implement the clinical supervision model (CSM). During the implementation of the CSM, the most common issues arise regarding the synchronous delivery of remote lessons by teacher candidates, observation of these lessons by supervisors, and the subsequent feedback provided. Additionally, observing friends and cooperating teachers delivering lessons is also a concern. As conducting practicums in a digital environment is a relatively new phenomenon, there are limited studies on how preservice teachers are affected by this process. The studies have concluded that teacher candidates feel inexperienced and incompetent due to the fact that the practicum course cannot be carried out face-to-face in practicum schools, which negatively affects their professional competences [14].

Teacher training programmes that have transitioned to distance education due to the pandemic must be adapted to current needs and living conditions [15]. To achieve the intended goals of the practicum, the clinical supervision model, an essential component of the practicum process, must also be integrated into distance education. A new clinical supervision model is required that is adaptable to existing living conditions and can be modified as these conditions change. The model should facilitate easy and speedy adaptation to educational conditions. An effective clinical supervision process can be achieved through the implementation of a hybrid flexible (HyFlex) education approach.

The HyFlex course design model was first introduced and developed by Brain J. Beatty in 2005 [16]. HyFlex is an approach that offers students the opportunity to learn in three different modes: face-to-face, remote synchronous, and asynchronous. HyFlex courses provide students with the flexibility to customize their learning experiences by blending online and in-person components, following the guidelines set by the instructor. The HyFlex model enables students to choose how they participate in the course and create their own blend of online and face-to-face learning experiences, within the design constraints set by the instructor.The HyFlex course design is based on four fundamental principles: Learner Choice, Equivalence, Reusability and Accessibility. That is, instructors should provide meaningful alternative modes of participation and allow students to choose between daily, weekly or topical modes of participation (Learner Choice) and provide equivalent learning activities in all modes of participation, resulting in equivalent learning (Equivalence). Different materials and activities used in teaching should be converted into files and formats that are accessible to both online and face-to-face learners. And these materials should also be converted into permanent learning resources for all students in future courses (Reusability). Also, students should be equipped with technology skills and necessary technology such as internet connection, hardware, software, computers to access all modes of participation so that all course materials and activities should be accessible and useable by all students (Accessibility) [[16], [17]].

• LMS-based HyFlex Clinical Supervisory Model (HyFlex CSM)

In the LMS-based HyFlex educational approach, students have the flexibility to choose between traditional face-to-face education and remote synchronous education, with the option to switch between these modalities as needed. In addition, students have the option to choose asynchronous online learning for a specific activity during a given week, and they can choose different learning formats for the same activity in subsequent weeks [17]. The HyFlex education model is student-centric, allowing students to make weekly decisions based on their individual circumstances. The HyFlex model has been shown to be particularly useful in addressing unforeseen challenges, such as illness, transportation issues, unexpected caregiving needs, or other unique situations. Studies on courses that have implemented this model have reported high levels of student satisfaction [18], active course participation [19,20], and academic performances similar to those of traditional instruction [21].

The HyFlex CSM proposed in this study consists of three main cycles through the Learning Management System (LMS). In the ‘Before’ cycle, teacher candidates can choose to receive feedback on their draft lesson plan in three modes: face-to-face, synchronous, and asynchronous. Face-to-face feedback can be oral or written, with oral feedback provided during lesson breaks and written feedback delivered through platforms such as WhatsApp or email. Alternatively, teacher candidates have the option to receive remote synchronous feedback via Zoom or Google Meet. Asynchronous feedback may involve previously recorded audio, video, or document/PDF/image files uploaded to the LMS. During the ‘While’ cycle, the teacher candidate conducts the lesson face-to-face in the actual classroom, with cooperating school mentor and university mentor, as well as groupmates, observing either face-to-face or remotely synchronously through interactive platforms like Zoom. In the ‘After’ cycle, feedback sessions take place either face-to-face or remotely, synchronously or asynchronously. Asynchronous feedback is provided via the LMS, which includes mentors' pre-recorded audio (MP3) or visual (MP4) files of Zoom or Google Meets, along with the uploading of observation notes, written feedback, and observation forms to the LMS.

The studies consistently highlight that the abrupt transition from traditional face-to-face teacher practicum to remote learning, due to the pandemic, caught teacher candidates off guard and had negative effects on them. Given the existing research that primarily highlights the challenges and drawbacks of online practicum, it is increasingly important to adopt a problem-solving-oriented approach in the research landscape. The focus should be redirected towards proposing strategies for the effective implementation of online practicum. These strategies should be enduring rather than temporary, considering the possibility of other pandemics or pandemic-related scenarios.

The objective of this study is to introduce the Hyflex clinical supervisory model, which can be used for both face-to-face and remote teacher practicums. The study aims to identify the preferences of teacher candidates for different modes of activity delivery, such as lesson observation, teaching sessions, and feedback, within the context of this comprehensive model during their practicum experiences.

Knowledge of which activities in the HyFlex mentoring process are preferred in each mode will provide valuable information on how to reshape the traditional clinical supervisory model for more effective implementation. The COVID-19 pandemic has led to a transition from face-to-face to remote learning at all levels of education. Many educational institutions faced challenges during the transition to remote learning due to inadequate infrastructure. This study proposes a mentoring model that can facilitate the transition to remote education during unexpected circumstances, such as the COVID-19 pandemic. The data obtained on preferences for activity modes in the HyFlex mentoring process will provide valuable insights into how to reshape the traditional clinical supervisory model to be more effective, especially in times of unforeseen challenges such as the COVID-19 pandemic.

The purpose of this study is to investigate the mode preferences of teacher candidates regarding the activities carried out during the clinical supervisory process. The research questions guiding this study are as follows:

1) What are the preferred modes of teacher candidates for receiving feedback on their lesson plans before teaching sessions?
2) What are the preferred modes of teacher candidates for constructing teaching sessions?
3) What are the preferred modes of teacher candidates for observation?
3a) What are preferred modes of teacher candidates for being observed by mentors and groupmates?
3b) What are the preferred modes of teacher candidates for observing the cooperating school mentor?
4) What are the preferred modes of teacher candidates for feedback after teaching sessions?

## 2 Materials and methods

### 2.1 Research design and sampling

The research adopts a mixed methods design, using both qualitative and quantitative approaches to collect data concurrently. Two primary data collection methods, a questionnaire and interviews, were used to explore the research objectives. Snowball sampling was used to expand the participant group through individuals or groups initially approached by the researcher. In order to expand the sample, potential participants are reached through a few identified participants. The study was conducted during the period of quarantine caused by the COVID-19 pandemic. Teacher candidates were contacted through social communication networks such as WhatsApp and Facebook. They were asked to inquire whether any teacher candidates in their placement groups were interested in participating in this study on a voluntary basis. Those who responded were also asked to seek the support of their colleagues in the ELT department who might also be interested in participating. A total of 58 students studying and interning in ELT departments in different regions of Turkey were reached through this process.

### 2.2 Participants

The study involved 58 4th year teacher candidates from the ELT departments of six different state universities located in five regions of Turkey: Eastern Anatolia, Marmara, Mediterranean, Central Anatolia and Black Sea regions. Of the 58 teacher candidates, 43 were female and 15 were male. Of these, five male and seven female teacher candidates volunteered to be interviewed.

### 2.3 Data collection tools, data collection procedures and data analysis

#### 2.3.1 Questionnaire

To collect data for the study, a questionnaire was developed, comprising two main parts. The first part focuses on demographic information, while the second part gathers data on teacher candidates' perceptions of different modes in the mentoring process. The second part is further divided into sub-categories: “Before Teaching Sessions,” “While Teaching Sessions,” and “After Teaching Sessions."

To ensure internal validity, the survey was reviewed by two field experts. A consensus was reached based on objective evaluations that all topics related to the clinical supervisory process and mentoring during the internship period were comprehensively covered. The questions were confirmed to be clear and understandable. To ensure face validity, the survey was administered to four volunteer teacher candidates to identify any unclear or ambiguous statements in the questions or instructions. Corrections were implemented based on suggestions received during the pilot study, resulting in the final version of the online survey created using Google Forms.

During data analysis, participant responses for the options ‘strongly agree’ and ‘agree’ were combined to reflect a positive approach to the respective question item. Similarly, the options ‘strongly disagree’ and ‘disagree’ were merged to express a negative sentiment. The table presents the consolidated values of participant responses, including the original form of the ‘undecided’ option.

#### 2.3.2 Interview

For the Google Forms survey, participants who indicated their willingness to be interviewed were requested to provide their email address. Zoom or Google Meetings were scheduled with the volunteers. The interviews were conducted in Turkish, recorded, and transcribed into text using Microsoft Word. The semi-structured interviews provided qualitative data that broadened the perspective of the research questions and facilitated a more comprehensive interpretation of the results obtained from the quantitative survey. This was achieved by incorporating interview quotes into the findings of the relevant research questions. The interview questions align with the research objectives.

1) What are your preferred modes for receiving feedback on your lesson plans before teaching sessions? Why?
2) What are your preferred modes for constructing teaching sessions? Why?
3) What are your preferred modes for observation? Why?
3a) What are your preferred modes for being observed by mentors and groupmates? Why?
3b) What are your preferred modes for observing the cooperating school mentor? Why?
4) What are your preferred modes for feedback after teaching sessions? Why?

#### 2.3.3 Ethics

A consent form outlining the study's purpose, participant involvement, and the intended use of collected data was presented to participants on the first page of the online questionnaire. They were explicitly informed about the research, assured of anonymity and confidentiality, and provided with the researcher's contact information. Similarly, a distinct yet similar informed consent form was prepared for participants engaging in online interviews via Zoom. The study received approval from the Institutional Review Board (Ethics Committee) of Inonu University (Identification code E.412079), adhering to ethical principles.

## 3 Results

Tables presenting the relevant sections of the survey for the four research questions in this study, including calculations and corresponding percentages, are provided. It is important to note that questions 6, 9, 10, 11, and 29, along with their corresponding answers, have been excluded from the survey prepared on Google Forms. The item numbers have been kept unchanged to maintain the originality of the survey.

### 3.1 Research question 1: what are the preferred modes of teacher candidates for receiving feedback on their lesson plans before teaching sessions?

While preferences for face-to-face and asynchronous feedback seem opposed, the commonality is the auditory nature of feedback. Teacher candidates indicated a preference for audio feedback over written feedback. The findings show high acceptance rates for various feedback modes, with asynchronous leading at 59.6 % (Item 4), face-to-face at 50.0 % (Item 1), and synchronous at 43.1 % (Item 3) (Table 1 a).

**Table 1a:** Mode preference rates of teacher candidates for receiving feedback on their Lesson Plans.

Item no	Item	Strongly disagree (1) + Disagree(2)	Neutral	Strongly agree (5) + Agree (4)
1	I prefer to receive feedback on my lesson plan through face-to-face discussions	25,8	2,41	50,0
2	I prefer to receive feedback on my lesson plan through remote synchronous meetings (such as simultaneous Zoom meetings) before my teaching sessions	27,6	29,3	43,1
3	I prefer to receive feedback on my lesson plan remotely through asynchronous (written) means (such as notes taken on paper or forms that can be uploaded to the LMS).	29,3	27,6	43,1
4	I prefer to receive feedback on my lesson plan remotely through asynchronous (audio) means (such as uploading feedback in the form of voice messages to the LMS).	24,1	17,2	59,6

The results of the backup question (Item 5) indicate that 47.4 % of candidates prefer a hybrid mode for feedback before teaching sessions, which includes audio and face-to-face modes. This suggests a strong preference for flexibility in choosing feedback modes (Table 1b).

**Table 1b:** Mode preference rates of teacher candidates for receiving feedback on their lesson plans.

Item no 5:Which mode do you prefer for discussing your lesson plan with your mentor before the teaching session and receiving feedback on it?	Frequency (%)
Face to face	15,8
Remote synchronous (video conferencing/Zoom meeting)	15,8
Asynchronous (written): I prefer my mentor to upload the written feedback document to the LMS.	8,8
Asynchronous (verbal): My mentor can leave an audio message on the LMS, or upload a video message file with both audio and video components to the LMS.	12,3
Hybrid: I would like the option to choose different modes depending on the situation	47,4

The importance of asynchronous feedback was highlighted by a teacher candidate, who expressed a preference for both audio and written feedback due to its permanence.

“ Feedback is typically asynchronous for us. This approach has benefits because when we are with the instructor (mentor), we may forget to take notes on certain topics or miss important information. Missing verbal feedback can have a significant impact on the lesson plan.." (M2)

Another teacher candidate emphasized the advantage of asynchronous feedback, especially for those away from the regular setting:

“ I think that face-to-face interaction is not necessary. Instead, tasks can be completed asynchronously, such as uploading the lesson plan to the LMS and receiving feedback. For example, a mentor may provide feedback in the form of written notes or a voice recording. Asynchronous communication is more convenient for everyone, especially when I am out of town. “(M3)

The second most preferred feedback mode was face-to-face (50 %). A teacher candidate choosing this option valued the immediacy for asking questions and receiving instant answers:

“I prefer face-to-face. Because I can get an instant response to my question when face-to-face. This could be in the format of a conversation over Zoom or a direct physical conversation. But the immediacy is more important for me." (F1)

Another teacher candidate emphasized the importance of ‘gesture’ during feedback sessions:

“I prefer to receive my feedback verbally. It's nicer face-to-face; we receive feedback from our mentor either through Zoom or phone calls. However, sometimes it's difficult to fully understand the teacher's message with just voice or text. Body language is necessary to comprehend the person in front of you. Face-to-face feedback is more helpful to me.” (M3)

### 3.2 Research question 2: what are the preferred modes of teacher candidates for constructing teaching sessions?

When asked about their teaching preferences, a significant majority of teacher candidates (82.8 %, Item 7) expressed a preference for face-to-face instruction (Table 2a).

**Table 2a:** Mode preference rates of teacher candidates for teaching sessions.

Item no	Item	Strongly disagree (1) + Disagree(2)	neutral	Strongly agree (5) + Agree (4)
**7**	I prefer to deliver the lesson in a face-to-face real classroom environment	6,8	10,3	82,8
**8**	I prefer to deliver the lesson remotely through synchronous means (using virtual class platforms like Zoom).	39,7	25,9	34,5

A teacher candidate noted the benefits of in-person education, as it allows for the observation of students' gestures and facial expressions, enabling real-time adjustments to the lesson based on their reactions:

“*Honestly, I would have preferred face-to-face teaching as it would allow me to observe students' gestures and facial expressions. This way, I could bring them back to the lesson if I noticed they were getting bored. Unfortunately, I didn't have that opportunity and often couldn't tell when students were disengaged from the lesson. Upon reflection, when I thought about the lesson or watched the recordings,I realised that the children had disengaged from the lesson, either not listening or showing no interest, yet I continued to explain.. Therefore, my words were ineffective.”(*F1

The survey also revealed the hybrid mode as the second most preferred teaching mode (35.1 %, Item 17) (Table 2b). A teacher candidate, with limited experience in face-to-face teaching, stated a preference for the hybrid approach, citing student-related reasons:

“ To be honest, I didn't have much experience in a large classroom setting other than providing face-to-face private lessons. However, if it were a traditional face-to-face class, I would prefer a hybrid approach. This would accommodate students who may have physical discomfort or are not in a mentally sound condition on that day and cannot attend. They could listen to the recordings after the lesson. “(F1)

**Table 2b:** Mode preference rates of teacher candidates for teaching sessions.

Item 17: When delivering your teaching sessions, which mode do you prefer?	Frequency (%)
Face to face	45,6
Synchronous	19,3
Hybrid: I would like the option to choose different modes depending on the situation	35,1

### 3.3 Research question 3: what are the preferred modes of teacher candidates for observation?

#### 3.3.1 What are preferred modes of teacher candidates for being observed by mentors and groupmates?

During their teaching sessions, teacher candidates express varied preferences for observation by cooperating school mentor, university mentors, and groupmates. Notably, the highest preference is for not being observed by groupmates (17.7 %, Item 19). The hybrid mode of observation is most favored when it involves the university mentor (42.1 %, Item 20). Additionally, a higher preference is observed for face-to-face observation by cooperating school mentor compared to groupmates or university mentors (33.3 %, Item 18) (Table 3).

**Table 3:** Mode preference rates of teacher candidates for being observed by mentors and o groupmates.

**Item 18: When delivering your teaching sessions face-to-face, which mode do you prefer your cooperating school mentor to observe you?"**	Frequency (%)
Face to face	33,3
Remote synchronous (video conferencing/Zoom meeting)	12,3
I record my asynchronous teaching sessions and upload them to the LMS for viewing.	22,8
Hybrid: I would like the option to choose different modes depending on the situation	29,8
I do not want my cooperating school mentor to observe my teaching session.	1,8

**Item 19: When delivering your teaching sessions face-to-face, in which mode do you prefer your groupmates to observe you?**	
Face to face	28,1
Remote synchronous (video conferencing/Zoom)	15,8
I record my asynchronous teaching sessions and upload them to the LMS for viewing.	14,0
Hybrid: I would like the option to choose different modes depending on the situation	24,6
I do not want my groupmates to observe my teaching session.	17,5

**Item 20: When delivering your teaching sessions face-to-face, in which mode do you prefer your university mentor to observe you?**	
Face to face	17,5
Remote synchronous (video conferencing/Zoom meeting)	15,8
I record my asynchronous teaching sessions and upload them to the LMS for viewing.	21,1
Hybrid: I would like the option to choose different modes depending on the situation	42,1
I do not want my university mentor to observe my teaching session.	3,5

One teacher candidate favours a face-to-face observation by the cooperating school mentor, emphasizing the comprehensive feedback that is possible in a physical classroom environment.

“Your teacher can observe not only how you deliver the lesson but also your posture in the class, the authority you can establish, class discipline, and classroom management." (M3)

In contrast, those preferring remote synchronous observation by the university mentor cite nervousness as a factor:

“If my practice teacher (university mentor) were to observe me live, I would get very nervous. I might start doing things I wouldn't normally do. Even if I made eye contact with the teacher in the classroom (cooperating school mentor), I think I would still get nervous. But teachers observing me from a distance actually relax me more because I don't see their faces. Maybe they would frown, and I would understand, or if I saw the teacher's face, I would start watching them this time." (F2)

The most preferred mode of being observed by peers while teaching is face to face (28.1 %, item 19), with teacher candidates expressing that the presence of their group peers in the classroom relaxes and motivates them (Table 3). However, some teacher candidates stated in the interviews that they found it less stressful when their group mates observed them synchronously from a distance during their teaching sessions.

“ My friends didn't stress me out much. Sometimes, I even forgot they were there because there was no visual or auditory stimulus.” (F1)

#### 3.3.2 What are the preferred modes of teacher candidates for observing the cooperating school mentor and group mates?

A large majority of teacher candidates (77.6 %, Item 12) prefer to observe their mentors' lessons in person, within the classroom setting. In relation to the observation of groupmates' lesson presentations, over half of the teacher candidates (63.8 %, Item 14) prefer either face-to-face or remote synchronous modes. The majority favour synchronous observation at 51.8 % (Item 15) (Table 4).

**Table 4:** Mode preference rates of teacher candidates for observing the cooperating school mentor and groupmates.

	Item	Strongly disagree (1) + Disagree(2)	neutral	Strongly agree (5) + Agree (4)
**12**	I prefer to observe my cooperating school mentor face-to-face when teaching.	8,6	13,8	77,6
**13**	I prefer my cooperating school mentor to conduct live broadcasts using a simultaneous virtual class platform while teaching in the classroom. This way, I can observe him/her remotely and synchronously without physically going to the cooperating school.	15,5	37,9	46,5
**14**	I prefer to observe my group mate's teaching sessions in a face-to-face real classroom setting.	13,8	22,4	63,8
**15**	If my group mate conducts a live broadcast using a simultaneous virtual class platform while presenting in the classroom, I prefer to observe them remotely synchronously without going to the cooperating school.	19,0	29,3	51,8
**16**	Instead of observing my group mate's face-to-face teaching sessions in the cooperating school, I prefer to asynchronously watch the recorded and uploaded video.	27,65	31,0	41,4

One teacher candidate advocates for face-to-face or synchronous feedback while expressing reservations about asynchronous feedback. The candidate highlights the difficulties of monitoring and promptly accessing asynchronous content, resulting in reduced engagement.

“*I prefer face-to-face or live, synchronous communication. If we are in the same city, I would definitely choose face-to-face, which I have done before.As I mentioned, I prefer either face-to-face or synchronous communication. When it's asynchronous, I doubt that most teacher candidates will engage. The recorded lessons are too long to watch.As a student, I experienced this in some of my classes.Normally, we attended them synchronously, live. However, there was a teacher who provided asynchronous lessons, and honestly, I don't think anyone watched them. It's easy to keep postponing it. I did the same for my other university courses. The exam is approaching, and I still haven't reviewed the course materials, even though I said I would.”* (M3)

The results indicate a clear preference order for different observation modes of groupmates, from most to least preferable: face-to-face, synchronous, and asynchronous. The trend among teacher candidates suggests that face-to-face observation remains the most preferred mode.

### 3.4 Research question 4: what are the preferred modes of teacher candidates for feedback after teaching sessions?

Teacher candidates show a strong preference for various forms of mentor feedback, indicating their openness to different modalities. Notable preferences include face-to-face (60.3 %, Item 21), synchronous (55.2 %, Item 22), written asynchronous (46.6 %, Item 23), and audio/recorded video asynchronous (58.6 %, Item 24) feedback. The overall trend highlights the importance that teacher candidates place on receiving mentor guidance, regardless of the mode (Table 5a).

**Table 5a:** Mode preference rates of teacher candidates for feedback after teaching sessions.

Item no	Item	Strongly disagree (1) + disagree(2)	neutral	Strongly agree (5) + Agree (4)
**21**	After my teaching session, I prefer to receive mentor feedback face-to-face.	20,7	19,0	60,3
**22**	I prefer to receive mentor feedback after my teaching session through remote synchronous discussion during the day (a meeting session can be conducted via Zoom)	19,0	25,9	55,2
**23**	I would like to receive written feedback after my teaching session, preferably asynchronously.(My mentor can upload the notes taken or observation form filled out during the session to the LMS.)	38,0	15,5	46,6
**24**	I would like to receive feedback remotely in real-time (verbal/visual) after my teaching session. (My mentor can upload the feedback to the LMS as an audio/visual message.)	20,7	20,7	58,6
**25**	Following the teaching session, I would like my peers who observed my teaching to attend the face-to-face feedback meeting held at the cooperating school.	22,4	24,1	53,5
**26**	Instead of having my groupmates attend the face-to-face feedback session with the mentor after my teaching session, I prefer them to watch the recorded feedback session that is uploaded to the LMS.	34,5	25,9	39,7
**27**	There is no objection to my groupmates who observe my teaching session having access to the written/verbal feedback uploaded to the LMS by my mentor.	26,8	32,1	41,1

In relation to groupmates' participation in feedback sessions, more than half of the teacher candidates (53.5 %, Item 25) view face-to-face engagement positively (Table 5a). This collaborative approach is perceived as a chance for mutual learning and improvement, as expressed by one teacher candidate:

“They can join as well. When entering the lesson with my peers, the area where I lacked was time management. Many of my groupmates also struggle with time management. If they give me feedback on this issue, my mentor and those friends can listen to those feedbacks and turn them to their advantage. This could be a nice aspect. Now that we are teachers, the goal during this process should be to receive valuable feedback and use it to improve. That's why I think it would be selfish for teacher candidates to only receive individual feedback." (M1)

Despite being open to various feedback modalities, 60.4 % (Item 26) of teacher candidates are hesitant to allow their groupmates access to their recorded feedback sessions uploaded to the LMS. This indicates a reluctance to share feedback sessions, whether synchronous or asynchronous (Table 5a).

Almost half of the teacher candidates (46.6 %, Item 28) indicated a preference for a combination of feedback modalities, making the hybrid feedback mode a popular choice (Table 5b). This suggests that candidates have a nuanced understanding and desire mentor guidance tailored to their individual preferences and needs.

**Table 5b:** Mode preference rates of teacher candidates for feedback after teaching sessions.

Item 28: Following your teaching session, in what format would you prefer the feedback meeting on your teaching?	Frequency (%)
Face to face	24,6
Remote synchronous (feedback meeting over Zoom during the day).	17,6
Asynchronous (written): Uploading written feedback documents to the LMS (Learning Management System).	8,8
Asynchronous (verbal/visual): Uploading audio/visual messages to the LMS (Learning Management System)	3,5
Hybrid: I would like the option to choose different modes depending on the situation	46,6

However, a teacher candidate expressed a preference for receiving face-to-face feedback from the cooperating school mentor in a Zoom session after teaching, emphasizing the importance of timing for this feedback.

“I honestly don't want it right after teaching because I get tense after teaching. I can misunderstand or not perceive some feedback. For me, it would be more comfortable to have a Zoom session with my mentor in the evening when I'm in a calm state." (F2)

Notably, the hybrid mode is the most preferred feedback mode, highlighting the significance of flexibility and personalized approaches in the feedback process. According to Table 5b, only 24.6 % (Item 28) of teacher candidates preferred face-to-face feedback. This highlights the diverse and evolving landscape of feedback modalities.

## 4 Discussion

### 4.1 Mode preferences for feedback

In clinical supervision, ‘reflection’ involves self-awareness, critical reflection, learning from experience, continuous improvement, and the application of theory to practice. Teacher candidates explore their thoughts and emotions, analyse experiences deeply, and connect their experiences to relevant theories for a more comprehensive understanding of their professional practice [22]. In their work, [23] introduces the concepts of ‘reflection-in-action’ and ‘reflection-on-action.’ In the context of this research, ‘reflection-on-action’ takes place through synchronous feedback conferences held after teaching sessions, involving both teacher candidates and mentors. The study's findings indicate that teacher candidates are receptive to a mix of mentor feedback types, highlighting the importance they place on receiving mentor guidance tailored to their personal preferences and requirements after their teaching sessions. The results are intriguing because despite teacher candidates not having any traditional face-to-face communication with mentors or groupmates during their internships, all mentor feedback modes were equally preferred.

As a result of the lockdown, teacher candidates were required to conduct their practicum online, which led to a separation from their mentors and groupmates. This forced the candidates to work in isolation, making it particularly important for them to receive support and feedback regarding their professional competencies. Based on the findings of this research, teacher candidates value both synchronous and asynchronous feedback received after teaching sessions. They believe that any form of feedback from mentors would be beneficial for their professional growth. This is consistent with previous research which suggests that the effectiveness of online mentoring depends on how teacher candidates perceive the support and input provided by mentors for their professional development [24].

[22] emphasizes the importance of immediate, personal interactions to enhance professional learning. In the present study, one of the teacher candidates instantly mentioned that he preferred to receive feedback sometime after his teaching session, as he feels stressed just after the teaching session and wants to be mentally ready. The teacher candidate's choice of synchronous delayed feedback may be attributed to his belief that engaging in self-reflection before the synchronous feedback conference with the mentor enhances the effectiveness of the session. Delayed feedback could be in the form of either synchronous or asynchronous forms. Asynchronous communication, marked by delayed communication [25] which is also referred to as asynchronous computer-mediated communication (CMC) by [26] offers a flexible approach for both university mentors and cooperating school mentors to provide tailored guidance to teacher candidates. This form of feedback can take diverse formats, such as written or audio comments on lesson plans, personalized insights conveyed through recorded audio messages, and comprehensive mentor feedback reports that pinpoint specific areas of strength and areas for improvement, which can also be facilitated through online platforms, allowing mentors to offer guidance and recommendations at the candidate's convenience. [26] emphasize that such feedback is advantageous compared to traditional face-to-face feedback, as it allows for carefully composed messages without the pressure of immediate responses—contrasting the real-time dynamics of face-to-face discussions. In the same line, [24] indicate that asynchronous written feedback provides a more contemplative and task-oriented interaction compared to face-to-face discussions. This characteristic grants additional time for thoughtful responses, contributing to a reflective and effective mentoring process. In this study, asynchronous feedback, particularly in the form of mentor uploading audio feedback to the Learning Management System (LMS) for teacher candidates' lesson plans, was identified as the most preferred mentor feedback mode. One reason for this preference could be that teacher candidates, who have experienced both synchronous and asynchronous feedback from mentors, find asynchronous delayed feedback more content-based, aligning with the findings of [24,26]. Half of the students not preferring face-to-face mentor feedback may be due to concerns about the time and effort required to travel to the cooperating school or university to meet with mentors. [27] reports that a prevalent factor contributing to the failure of mentoring is the existence of time and place constraints. Online mentoring, including both synchronous and asynchronous mentor feedback in this context, offers increased flexibility compared to traditional face-to-face mentoring, as it is not bound by specific time or location constraints.

The results regarding teacher candidates' preferences for mentor feedback modes for both pre-teaching lesson plans and post-teaching session feedback indicate that asynchronous written mentor feedback is the least preferred in both cases. This preference may be due to the perceived lack of richness in content in written feedback provided by mentors. From the perspective of mentors, providing written feedback to teacher candidates can be challenging and time-consuming. Audio feedback was found to be pedagogically sound, practical only if necessary technological training provided and more holistic in terms of content [28].

Some teacher candidates indicated a preference for the synchronous mode for feedback after a teaching session, possibly because they may encounter difficulties in arranging a suitable time to convene for reflective conversations with the other triad members [29].

Most of the teacher candidates were positive about their peers' engagement in face-to-face discussions at the cooperating school, stating that this will enable them to learn from each other's mistakes. Similarly, the majority of the teacher candidates in [30] indicated the value of sharing experiences. In the same line, [31] investigated teacher candidates' field placements and found that, compared to teacher candidates placed alone, those placed in pairs learned more from their mentors. This collaborative exchange allows them to gather fresh insights applicable to their classrooms or contemplate aspects they should avoid. The positive attitude of teacher candidates towards engaging in face-to-face discussions with peers at the cooperating school, with an emphasis on learning from each other's mistakes, reflects the collaborative and social nature of reflective practice. [23] highlights the significance of shared experiences and mutual learning in professional development. The collaborative exchange described in the study corresponds to Schon's idea that professionals, through dialogue and interaction, can gain fresh insights applicable to their own practices or contemplate aspects they should avoid. This collaborative learning process is integral to Schon's concept of a reflective community where practitioners collectively contribute to each other's growth and development.

However, some of the teacher candidates were negative about the presence of their groupmates during the reflective conversation sessions. Similarly, [32] found that most of the teacher candidates showed negative response towards peers’ feedback, stating they provide unnecessary criticism. This unfavorable outlook may also be associated with concerns about privacy, as certain students might have personal questions and issues they prefer not to disclose during group meetings [33,34].

Highlighting the importance of teacher candidates receiving high-quality feedback for their professional development [35,36], another possible reason for this preference could be the desire for a more formal, constructive, and high-quality feedback environment provided by mentors. Teacher candidates may perceive their groupmates as being in a similar developmental stage compared to their mentors, which could lead to concerns about the quality and depth of feedback they receive. Additionally, groupmates might not offer the same level of expertise or provide feedback in as organized and detailed a manner as mentors. On the contrary, mentors, typically possessing greater experience and expertise, are considered valuable sources of insightful and structured feedback. Teacher candidates may perceive mentor feedback as more authoritative, drawing on the mentor's wealth of experience and pedagogical knowledge. The formality and guidance provided by mentors in the feedback process may be seen as crucial for professional growth and development.

### 4.2 Mode preferences for teaching sessions

During teaching sessions, teacher candidates expressed a preference for face-to-face interaction. Only 34.5 % of them favored delivering lessons remotely through synchronous means, utilizing virtual class platforms like Zoom. It is worth noting that the teacher candidates in this study lacked previous experience with in-person practicum opportunities. However, their preference for face-to-face instruction as the primary mode of teaching may be due to their lack of teaching experience and feeling unprepared for online teaching, as noted in previous research [[37], [38], [39], [40], [41]]. During the remote practicum period, students may have faced challenges outlined in the literature. The literature reports that teacher candidates commonly face challenges in classroom management [42,43], teaching methods and techniques [44], recognizing students' individual differences [43], engaging students in the lesson [45], technology-related issues [33,42], and inadequate interaction among teacher candidates or mentors [[2], [46], [47]].

However, it is important to note that a significant number of teacher candidates also expressed a preference for a hybrid mode, indicating an awareness of the potential benefits provided by a combination of in-person and remote components. The literature suggests that teacher candidates have experienced both benefits and challenges during online practicum [48].

In the present study, some teacher candidates reported facing difficulties in engaging students in the lesson, attracting their interest, sustaining their interest throughout the lesson, and managing the classroom effectively due to the inability to establish eye contact with students. However, other teacher candidates had a more positive experience with online practicum. It is important to note that these evaluations are subjective and should be clearly marked as such.

### 4.3 Mode preferences for observation

#### 4.3.1 Observing cooperating school mentor and group peers

One significant finding from the study is that teacher candidates have a clear preference for face-to-face observation for both their cooperating school mentor and group peers. This preference is likely due to the perceived benefits of in-person practicum, particularly in critical areas such as classroom management, as opposed to its online counterpart [40]. This inclination is supported by research indicating that teacher candidates consider face-to-face experiences indispensable, particularly in crucial aspects such as classroom management [40,48]. They may find it challenging to observe elements such as classroom management or grasp subtleties like non-verbal cues, class dynamics, and classroom management during lessons conducted via Zoom or other online platforms - integral components of successful teaching.

The preference for face-to-face observation among teacher candidates can be attributed to several factors. Firstly, teacher candidates seem to recognize the intrinsic value of collaborative peer observation. Of the three types of peer review (evaluative, developmental, and collaborative), the collaborative approach is considered the most effective. This is because it involves a cooperative nature, which helps minimize resistance from the individual or entity being reviewed [49]. Teacher candidates engage in observing, sharing insights, and discussing each other's teaching practices to promote self-reflection and mutual learning. The focus is on providing constructive feedback and fostering a positive and growth-oriented environment. This collaborative process benefits both observers and those being observed, contributing to continuous improvement in teaching skills. Furthermore, peer observation improves cooperation skills [50] and promotes intensive discussions [31,51]. It also fosters a sense of safety during teaching sessions [52] and provides opportunities to exchange collective experiences and teaching strategies [53]. Additionally, it offers psychosocial support [54,55].

It is important to note that the feedback provided is constructive rather than judgmental, which helps to foster a positive atmosphere [56]. In this study, teacher candidates who had not previously observed their cooperating school mentors or group peers in a real classroom may believe that face-to-face observations in live, natural classroom settings will provide them with enriched learning experiences. One possible reason for teacher candidates' preference for face-to-face observation is their belief in the importance of reflective practice. Reflective feedback sessions take place after observing each peer's teaching session, helping teacher candidates develop critical thinking skills and gain a deeper understanding of the observed teaching session. Observing a class in its natural environment allows teacher candidates to establish more meaningful connections with their notes, facilitating critical analysis of their teaching experiences. Using these notes during post-teaching discussions becomes more seamless, fostering a deeper and more nuanced understanding of their teaching practices, leading to a richer outcome in the post-teaching reflective meetings.

#### 4.3.2 Being observed by cooperating school mentor, university mentor and group peers

The results indicate that teacher candidates have a moderate preference to avoid being observed by their groupmates during teaching sessions (Item 19, 17.7 %). However, they have higher preference rates for being observed by their cooperating school mentors and university mentors. This less favourable outlook of teacher candidates may be associated with a nuanced interplay of psychological and social dynamics within the teacher candidate cohort.

One possible reason for this preference may be due to the perceived vulnerability that teacher candidates experience during their teaching sessions. The hesitancy to be observed by peers, especially those within the same developmental stage, may arise from a desire to present a flawless and polished image. Teacher candidates may be concerned that the presence of peers in the observation process could reveal imperfections or areas for improvement, potentially challenging the carefully curated image they wish to convey.

Moreover, the apprehension of judgment from peers may contribute to this unfavorable outlook. In a peer observation scenario, there might be a fear of being evaluated by individuals who share a similar level of expertise and experience. The prospect of receiving feedback or criticism from peers, even if constructive, could be perceived as more personally impactful than feedback from mentors. In line with this observation, [57] discovered that teacher candidates favored individual supervision over group supervision, echoing a similar reluctance found in the present study. This reluctance stemmed from their belief that peers might interpret their feedback as potentially negative. One possible reason for this preference could be the desire for autonomy among teacher candidates. This desire for independence is especially beneficial in single-field placements [51,[58], [59], [60]].

In the current research context, at the beginning of the practicum, teacher candidates are grouped into sets of six and assigned to a cooperating school mentor. Following the mentor's weekly lesson plan, teacher candidates are then organized in pairs, threes, or fours to coordinate their weekly observations and lesson presentations. In exceptional circumstances, such as when a candidate is employed or has conflicting university courses that clash with the internship group's schedule, the teacher candidate, with the approval of the university mentor, has the option to be grouped individually. However, the groups are typically in twos or threes, facilitating teacher candidates to observe each other's teaching sessions and participate in post-teaching feedback sessions.

When teacher candidates are placed in pairs or groups, they may feel that the classroom environment does not accurately reflect the realistic teaching environment they anticipate. They may be hesitant to take on the sole responsibility for the class while teaching in the classroom, and the presence of their peers could be seen as a potential challenge to that autonomy. In their study [51], compared the perceptions of teacher candidates in single field placements with those in group field placements. They discovered that the majority of teacher candidates regarded field placements as closer to reality. Additionally, in single field placements, candidates have more flexibility in preparing and instructing lessons as they do not divide or share teaching responsibilities with others [61].

## 5 Conclusions

Many studies have focused on the experience of virtual teaching practice during Covid-19 [[47], [62], [63], [64], [65], [66], [67]]. Different from these studies, the present research proposes to adapt the Hyflex approach to the traditional clinical supervisory mentoring and proposes a HyFlex Clinical Supervisory Model (HyFlex CSM) with three mode preferences for the activities such as teaching, observing and giving feedback (face-to-face, distance synchronous or asynchronous). By proposing such a new model, the study attempts to fill the gap in relation to the need for a model that supports the transition from a traditional teaching placement to a virtual one [68]. The HyFlex CSM programme provides teacher candidates with the opportunity to develop their skills in designing virtual lessons and materials, managing online learners, and efficiently utilizing technological tools and interactive learning platforms. In the event of an unexpected crisis, such as the Covid-19 pandemic, where complete closure is required, it may be more sustainable to use only remote modes rather than offering students three different mode choices. This will make the transition to full teaching placements much smoother for the triad members involved in the teaching practicum. The HyFlex CSM proposed here is not only applicable to ELT pre-service teacher education in Turkey, but also to other areas of pre-service teacher education worldwide. It is particularly useful for institutions that are uncertain about transitioning their conventional teacher education practicum from traditional face-to-face to a blended or fully virtual teaching format. Although the preferences identified by teacher candidates in the present study may reflect country-specific contextual preferences, they can be used as valuable information for future careful planning of the student teaching process for future unexpected disruptions such as Covid-19.

This model also takes into account the individual differences of teacher candidates and gives them the autonomy to choose from three different modes of delivery simultaneously: face-to-face, remote synchronous and remote asynchronous. This approach to instructional design is consistent with the principles of differentiated instruction, which recognises the unique learning characteristics of each student by offering flexibility in learning modes to accommodate different learning speeds, preferences and styles, resulting in a more personalized and effective learning experience for pre-service teachers.

Additionally, the HyFlex CSM contributes to an improvement in the quality of feedback provided to preservice teachers. Traditional clinical supervision can present challenges for university supervisors who may struggle to observe and provide feedback due to their busy schedules. This can make it difficult for them to travel to cooperating schools to observe teacher candidates and participate in post-feedback sessions. However, the HyFlex CSM addresses this issue by allowing for remote and synchronous observation. The HyFlex CSM is an innovative feature that overcomes the limitations of traditional supervision. It ensures that preservice teachers receive comprehensive and timely feedback, thereby enhancing the overall effectiveness of the mentoring process.

The current research is a preliminary step towards implementing this model in a real-time practicum. The members of the triad in the practicum setting include cooperating school mentors, teacher candidates and university mentors. This study focuses solely on the perspectives of teacher candidates regarding the potential application of this model. However, it is important to gather information about the mode choices of the other two members of the triad and to examine the differences and similarities in their preferences. It is crucial to understand the degree of agreement between the preferences of the triad members.

Therefore, studies examining the impact of the HyFlex CSM on the professional development of candidate teachers should also investigate the mode choices of all triad members. Examining mode choice is crucial to uncover the underlying reasons for these preferences. It is important to establish a methodological basis for mode choices to ensure that they are deliberate and not arbitrary.

The HyFlex CSM, with its multiple modes of instruction and adaptive feedback mechanisms, represents a paradigm shift in teacher practice. The implementation of the HyFlex CSM addresses traditional challenges and opens avenues for individualised learning. This innovative model for teacher education provides a resilient framework that is ready to meet the dynamic demands of evolving educational landscapes.

## Data availability statement

All data relevant to the study are included in the article.

## CRediT authorship contribution statement

**Ebru Melek Koc:** Writing – review & editing, Writing – original draft, Visualization, Validation, Supervision, Software, Resources, Methodology, Investigation, Formal analysis, Data curation, Conceptualization.

## Declaration of competing interest

The authors declare that they have no known competing financial interests or personal relationships that could have appeared to influence the work reported in this paper.
